# Second-Degree Burns in Neonates: A Rare Case Report of Saturation Probe Injury in Neonates

**DOI:** 10.7759/cureus.47761

**Published:** 2023-10-26

**Authors:** Kushal Desai, Amar Taksande, Revat J Meshram

**Affiliations:** 1 Paediatrics, Jawaharlal Nehru Medical College, Datta Meghe Institute of Higher Education and Research, Wardha, IND

**Keywords:** saturation probe injury, pulse oximetry, nicu, neonate, burns

## Abstract

Pulse oximetry is widely used in all intensive care units and in surgical monitoring and has the advantage of being noninvasive. Here, we report a 1.5 kg male neonate born via lower segment cesarean section at 5:00 pm. At birth, the patient had respiratory distress, mild subcostal retractions, minimal nasal flaring, and grunt audible with a stethoscope (Silverman-Anderson Score: 3) and was kept in the neonatal intensive care unit (NICU) with oxygen by heated humidified high-flow nasal cannula for observation of about 24 hours with a saturation probe connected to the right foot, due to which baby was found to have redness and swelling of the right foot with fluid-filled blebs on the palmar and dorsal surface in the morning at 8:00 am (18 hours of life), suggesting a second-degree burn.

Pulse oximetry is a noninvasive and basic test for seeing oxygen saturation but could lead to serious burn injuries, so special care must be taken by nursing staff and doctors to change the pulse oximeter site frequently as suggested in various case reports. Doctors and nursing staff need to be educated and made aware of the risk of pulse oximeter-related burn injuries.

## Introduction

Pulse oximetry is extensively used in all intensive care units and in surgical monitoring and has the advantage of being noninvasive [1]. It gives a real-time reading of arterial oxygen saturation and is the standard of choice [2]. Pulse oximeters have relatively high sensitivity (44%-76%) and high specificity (85%-90%) and give real-time information and, therefore, can be used in continuous monitoring in intensive care units and in operating theaters. The oximeter probe comprises a light source, which emits red and infrared light directly onto the skin, and a photodetector, which tracks how much light is absorbed by hemoglobin [3].

Chemical or thermal burns, tanning from the sensor light, pressure erosion, loss of sensation, and necrosis were among the complications related to using pulse oximetry. The exact causes of these problems are unknown. Still, they could be related to improper use of the probe, continued use, pressure ischemia caused by prolonged pressure on the same skin site, heating up of the probe, improper use of the probe, or overheating brought on by a short circuit in the probe cable [1,2].

Burns are uncommon; however, a few cases have lately been documented [3-5]. The pulse oximeter has been linked to burns through a number of processes. Electrical burn is brought on by current leakage from a damaged sensor's electrical components, chemical burn is brought on by a topical reaction to disinfecting agents contaminating the probe contact area, and thermal burn results from sensor heating up in a device that fails or prolonged duration of exposure. Furthermore, patients with diminished peripheral perfusion, babies with thinner skin, and advanced elderly individuals are also risk factors for burn injury [4].

The likelihood of a pulse oximetry injury may be influenced by the patient's health. Burn damage risk is increased by reduced blood flow to the location (typically a distal extremity) where the sensor is attached. Insufficient blood flow may prevent the heat produced by the light-emitting diode (LED) from leaving the area. As a result, the area becomes hotter and is exposed to heat for an extended period of time. This kind of harm can be caused by shock, hypothermia, or ischemia of the monitored extremity. The patient's thin skin makes more serious damage more likely. The skin on the toes and fingers, where sensors are typically located, may be thinner than on other body parts. The skin of neonates, especially those born prematurely, and the elderly is more frail and thin. They may also have inadequate peripheral perfusion. The sensor's compression of the skin may further reduce blood flow, which would make it harder for the body to expel heat produced by the sensor [5].

Neonates are quite distinct from older kids and adults in many ways. Management of neonatal burns presents difficult decisions because of their smaller size, skin that is thinner, more surface area-to-weight ratio, higher evaporative fluid losses, immaturity of the immune and renal systems, and different resuscitative needs because of their high daily fluid requirements per kg body weight [6]. Here, we present a case of a neonate who was kept in the neonatal intensive care unit (NICU) overnight for observation with a saturation probe connected to the right foot, due to which the baby was found to have redness and swelling of the right foot with fluid-filled blebs on the palmar and dorsal surface in the morning, suggesting a second-degree burn.

## Case presentation

**Figure 1:**
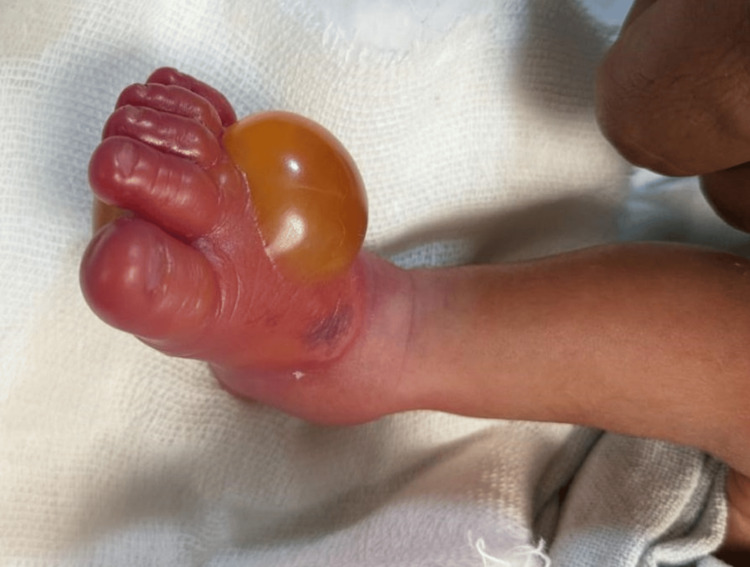
Right foot showing swelling and inflammation with a bleb on the dorsal part of the foot

**Figure 2:**
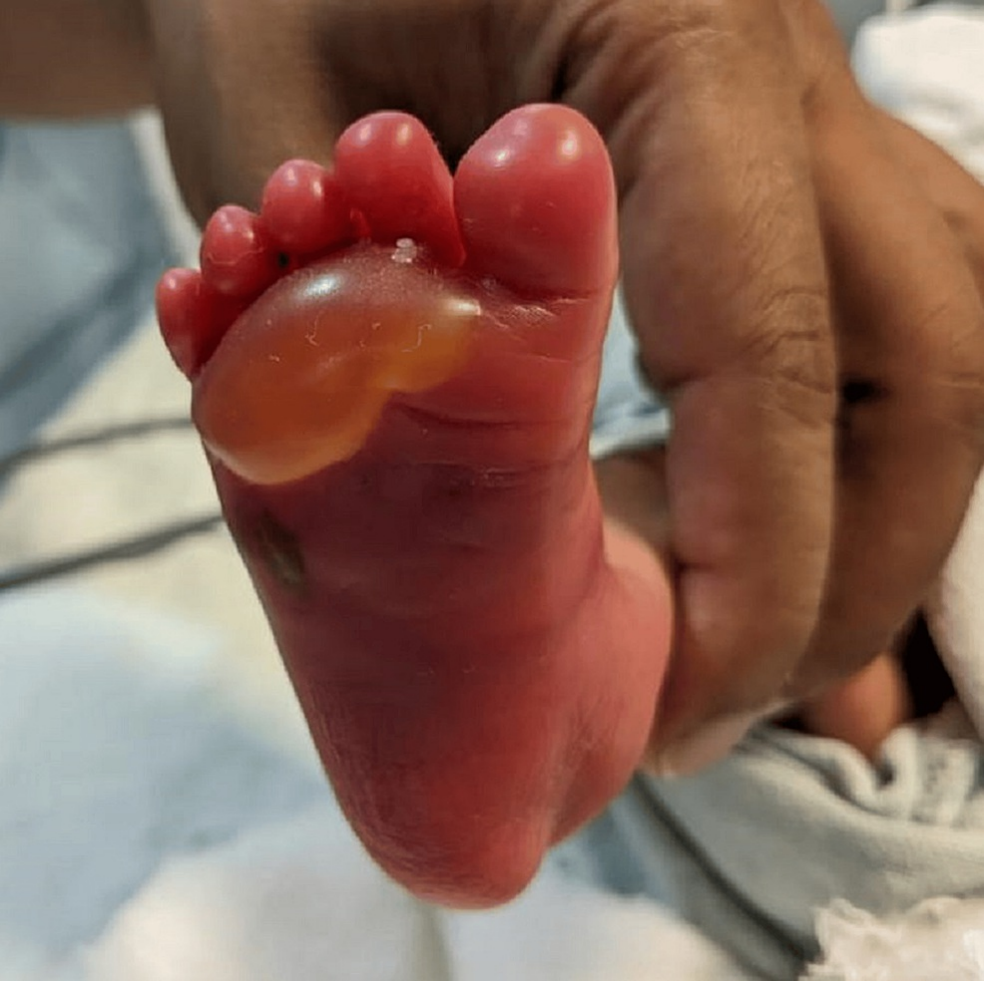
Right foot showing swelling and inflammation with a bleb on the palmar part of the foot

**Figure 3:**
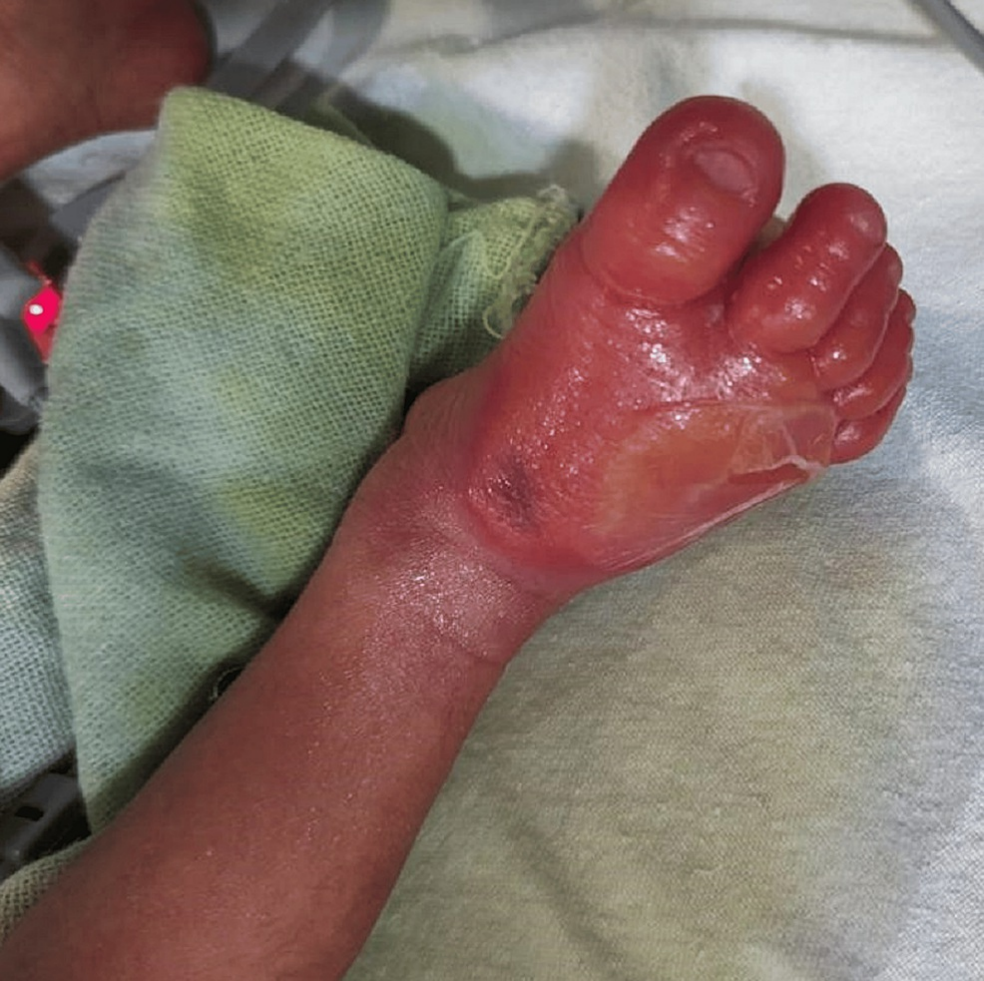
Right foot showing swelling and inflammation with the bleb burst on the dorsal surface as well as reduced swelling and redness

**Figure 4:**
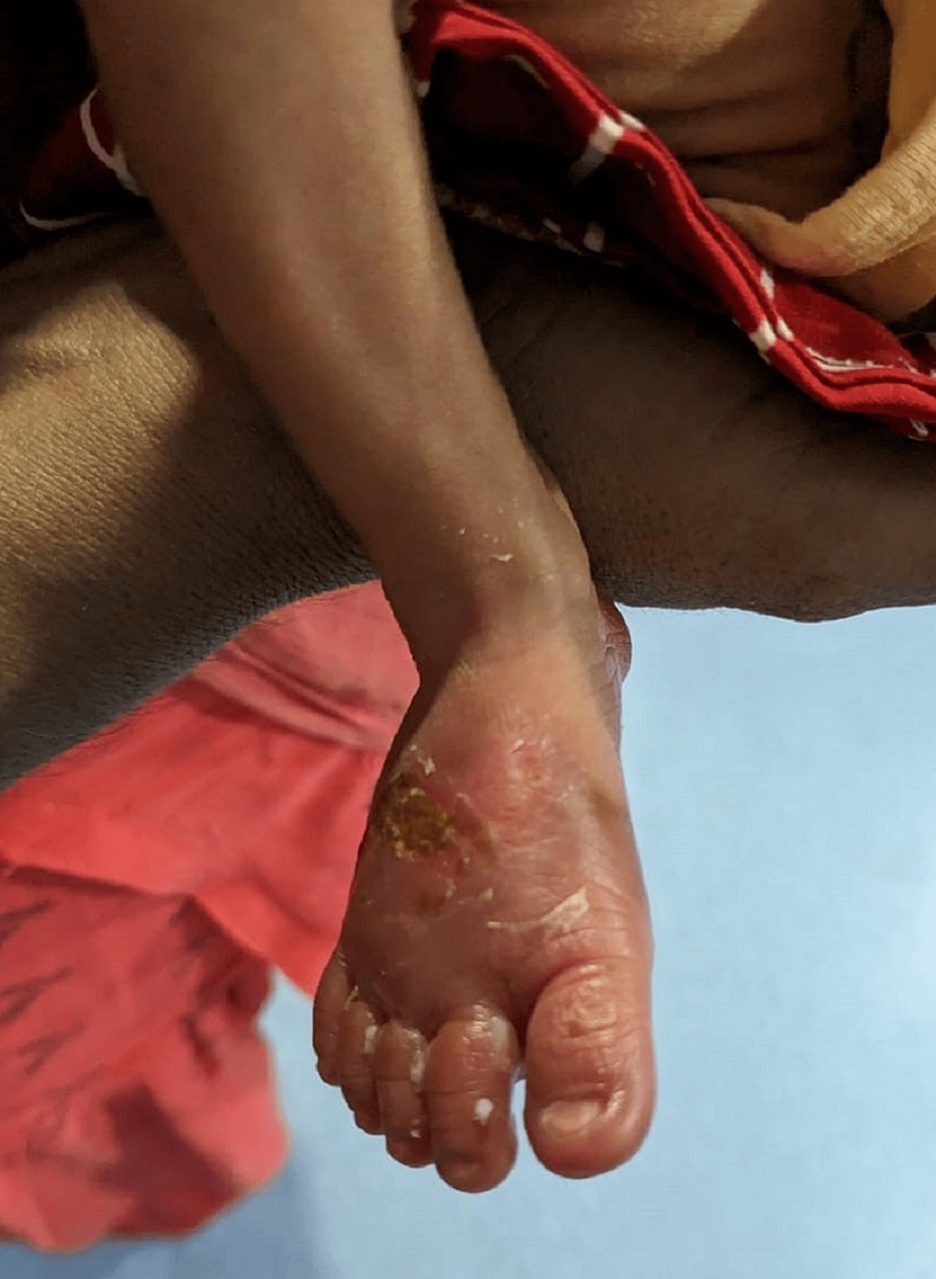
Right foot with reduced swelling and inflammation (picture on discharge)

A 1.5 kg male child was born to a G3P1L1A1 mother at 35.1 weeks of gestational age via normal vaginal delivery. The baby cried immediately after birth and had grunting heard without a stethoscope and mild nasal flaring, giving it a Silverman-Anderson score of 3. The baby was shifted to the NICU and kept on a heated humidified high-flow nasal cannula. Over two hours, grunting and nasal flaring subsided. The baby was weaned off the heated humidified high-flow nasal cannula, taken on oxygen by nasal prongs, and later weaned off oxygen. Katori spoon feed trial was given, which the baby accepted well. While on the heated humidified high-flow nasal cannula, an intravenous line was inserted on the left hand for fluids, baseline antibiotics (ampicillin and gentamicin) were started, and a septic screen was sent along with a complete blood count. The septic screen was negative, and complete blood count values were under normal values. The baby was kept in the NICU overnight for observation. The saturation probe was connected to the right foot. In the morning, while handing over to the sisters of the morning shift, the sister noticed that the baby had redness and swelling of the right foot (the saturation probe was in the right foot for about 10-12 hours) with fluid-filled blebs on the palmar and dorsal surface, suggesting a second-degree burn (Figures 1, 2). A pediatric surgery call was given, and a magnesium sulfate dressing was suggested with no further surgical intervention. The baby was started on paracetamol drops for pain and amoxicillin and clavulanic acid drops for decreasing inflammation. Later, the bleb on the dorsal surface burst itself. Silver nitrate cream application was advised. The baby was shifted to the mother's side after 72 hours, after the inflammation and redness reduced (Figure 3). The baby was discharged on the day of life 7 with advice to follow up within one week (Figure 4).

## Discussion

Pulse oximeters started being used in the 1980s and were considered to be safe and convenient, providing real-time arterial oxygen saturation. It was used initially by anesthetists in the operating rooms but now is widely used in almost all surgical and intensive care units. Burn injuries due to the probe have been reported, in which injuries range from discoloration of the affected limb, which is a mild complication, to various degrees of burns, which is a severe complication. The degree of complication is directly proportional to the time for which the probe position has not been changed [4]. Severe burns may cause full-thickness burns of phalanges where the pulse oximeter is attached and might cause gangrene and might need amputation, thermal burns in the setting of poor peripheral circulation, and extremity burns on infants with cases of gangrene and digit loss [7].

Greenhalgh et al. suggested that a temperature of up to 43°C for up to eight hours is safe for pulse oximetry probes [8]. In the present case, the neonate was kept in the NICU for mild respiratory distress (Silverman-Anderson score: 3) with a saturation probe connected to the right foot for about 10-12 hours with an unchanged position, due to which the baby was found to have redness and swelling of the right foot with fluid-filled blebs on the palmar and dorsal surface in the morning, suggesting a second-degree burn. Similar cases of such burns due to neonatal burns have been seen, but blister formation was rare.

The maintenance department was called to review and check the device's functioning and to check for faulty devices, but no fault was found. Therefore, frequently changing the pulse oximeter every 2-4 hours was tried. These methods are very small changes but are often overlooked. However, if overlooked, it can lead to severe manifestations, sometimes leading to amputation of the affected limb. Special care must be taken to neonates, elderly, and critically ill patients [4,5].

Most earlier burns produced by pulse oximeters that have been documented in the scientific community have resulted from inappropriate use or a problem with the device. According to reports, connecting sensor probes to displays made by different companies caused the sensors to overheat and cause thermal damage. In a case study by Murphy et al., it was shown that a high current flowing through the detector diode during equipment mixing can result in the tip temperature of the sensor spiking up to a great degree (117.2°C) in a short amount of time, approximately 3-5 minutes [9].

All types of burns were recorded in neonates, the most frequent being contact burns (29.5%), followed by flames (25.7%) and scalds (23.8%). Burns resulting from overexposure to radiating heat sources were encountered in 14.4% of cases, while chemical burns (4.8%) and electrical burns (1.9%) were rare.

Attaching the probe tightly to the extremity of a limb can decrease local blood flow to that area. This decreased blood flow due to pressure and temperature reduces heat dissipation and increases the chance of burns. The longer the probe is attached to the limb for monitoring, the more risk for burns and the more severe the burn [10]. There are various preventive techniques that could reduce the risk of burns, such as changing the probe site every 2-4 hours, loosely attaching the probe to the extremities adequate enough to get a proper reading, and avoiding using elastic adhesive tape for attaching probes [4,7,11]. Treatment of neonatal burns includes aggressive fluid resuscitation, keeping the patient warm, creation of a multidisciplinary team, early removal of necrosed tissue if any, application of specific biological dressing, and recombinant human growth hormone if necessary.

## Conclusions

Pulse oximeter monitoring is the cornerstone for monitoring arterial oxygen saturation in intensive care units worldwide. Burn injuries by pulse oximeters are quite rare, but if they occur, they are very severe and may cause limb gangrene, which may require amputation. Healthcare providers, doctors, and the nursing staff need to be aware of the risks of burns due to pulse oximeters, especially on neonates, and consciously try to avoid them.
